# Nitric oxide refines retinal circuit architecture independently of retinal wave dynamics

**DOI:** 10.1038/s41420-026-03228-2

**Published:** 2026-06-26

**Authors:** Lais Takata Walter, Marília Inês Móvio, Guilherme Shigueto Vilar Higa, Cayo Antônio Soares de Almeida, Reza Raeisossadati, Fernando da Silva Borges, Mariana Sacrini Ayres Ferraz, Giselle Cerchiaro, Christian Schmeltzer, Sten Rüdiger, Alexandre Hiroaki Kihara

**Affiliations:** 1 Centro de Matemática, Computação e Cognição, Universidade Federal do ABC, São Bernardo do Campo, SP Brazil; 2 Centro de Ciências Naturais e Humanas, Universidade Federal do ABC, Santo André, SP Brazil; 3 Institute of Physics, Humboldt University at Berlin, Berlin, Germany

**Keywords:** Cell signalling, Synaptic plasticity

## Abstract

How diffusible neuromodulators control neural circuit assembly remains a key open question in neuroscience. While nitric oxide (NO) is known to regulate mature synaptic plasticity, its role during development, specifically whether it shapes circuits through activity-dependent or activity-independent pathways, has not been fully understood. Here, we combine ultrasensitive electron paramagnetic resonance (EPR) spectroscopy, 4096-channel high-density multielectrode array (HD-MEA) recordings, and advanced graph-theoretical analysis to study how NO contributes to retinal network formation during a critical period of synaptogenesis. In the rat retina, nNOS expression begins at postnatal day 10 in two distinct amacrine cell subtypes, coinciding with the first detectable NO production. Acute or selective nNOS inhibition preserved the spatiotemporal features of Stage III retinal waves but significantly changed network topology, increasing network degree and density. Molecular profiling showed that nNOS blockade lowered the expression of chemical (SYN, SYP) and electrical (Cx36, Cx45) synaptic genes, disrupted their laminar distribution in vivo, and increased neurite length in primary retinal cultures without altering branching complexity. By combining ultrasensitive NO detection, large-scale electrophysiology, and mathematical network analysis, our results identify NO as a key regulator of circuit refinement that operates largely independently of the spatiotemporal dynamics of retinal waves during development. These findings reveal a molecular mechanism by which diffusible modulators shape neural networks independently of patterned activity and offer a framework for understanding how altered NO signaling might contribute to neurodevelopmental disorders characterized by impaired synaptic organization.

## Introduction

The formation and refinement of neuronal circuits during development rely on the precise orchestration of genetic programs and spontaneous neuronal activity [1, 2]. In the developing visual system, spontaneous activity patterns play a crucial role in guiding synaptic connectivity and the emergence of functional networks [3]. Nitric oxide (NO), a well-known modulator of synaptic transmission and plasticity in the mature brain [4, 5], has been implicated in developmental processes such as neuronal proliferation, survival, and differentiation [6]. However, the specific contribution of NO to the assembly of developing visual circuits, particularly within the retina, remains unclear.

The retina provides a robust model for investigating the mechanisms of circuit formation, as it generates stereotyped patterns of spontaneous activity known as retinal waves, which play a pivotal role in shaping retinofugal projections and local circuitry [7, 8]. Retinal waves evolve through three distinct stages characterized by their underlying neurotransmission mechanisms: in rodents, Stage I waves, mediated by gap junctions, emerge around embryonic day 17 and persist until birth [9, 10]; Stage II waves, mediated by cholinergic circuits, dominate from postnatal day (P)1 to P10 [11, 12]; and Stage III waves, driven by glutamatergic transmission from bipolar cells, coincide with the onset of robust synaptogenesis between P10 and P14 [13, 14]. Notably, this late stage of spontaneous activity temporally overlaps with critical periods of synaptic assembly, creating a window of opportunity for molecular modulators such as NO to influence network formation.

While NO has been shown to modulate topographic refinement in visual targets such as the superior colliculus and the dorsal lateral geniculate nucleus [15], its role within the retina itself remains unclear. In particular, it needs to be determined whether NO participates in shaping spontaneous activity patterns or whether it exerts its influence directly on synaptic organization and network connectivity. This gap is particularly relevant, given that alterations in NO signaling are increasingly linked to neurodevelopmental disorders characterized by impaired circuit formation.

In this study, we directly addressed these questions by combining high-density multielectrode array (HD-MEA) recordings, graph-theoretical analysis, gene expression profiling, and in vivo manipulations of NO signaling. Importantly, our results reveal that NO, primarily produced by nNOS-expressing amacrine cells during retinal development, is not essential for the generation and propagation of Stage III retinal waves. However, NO critically modulates the expression of synaptic genes, shapes the topological properties of neuronal networks, and regulates the spatial distribution of key synaptic proteins. Here, we show that NO produced by nNOS-expressing amacrine cells refines retinal circuit architecture without significantly altering the spatiotemporal properties of spontaneous retinal waves, revealing a mechanism of circuit organization that is dissociable from wave dynamics.

## Results

### Gene expression of NOS isoforms increases during development and accumulation of NOS isoforms follows distinct spatial patterns during retinal development

**Fig. 1:**
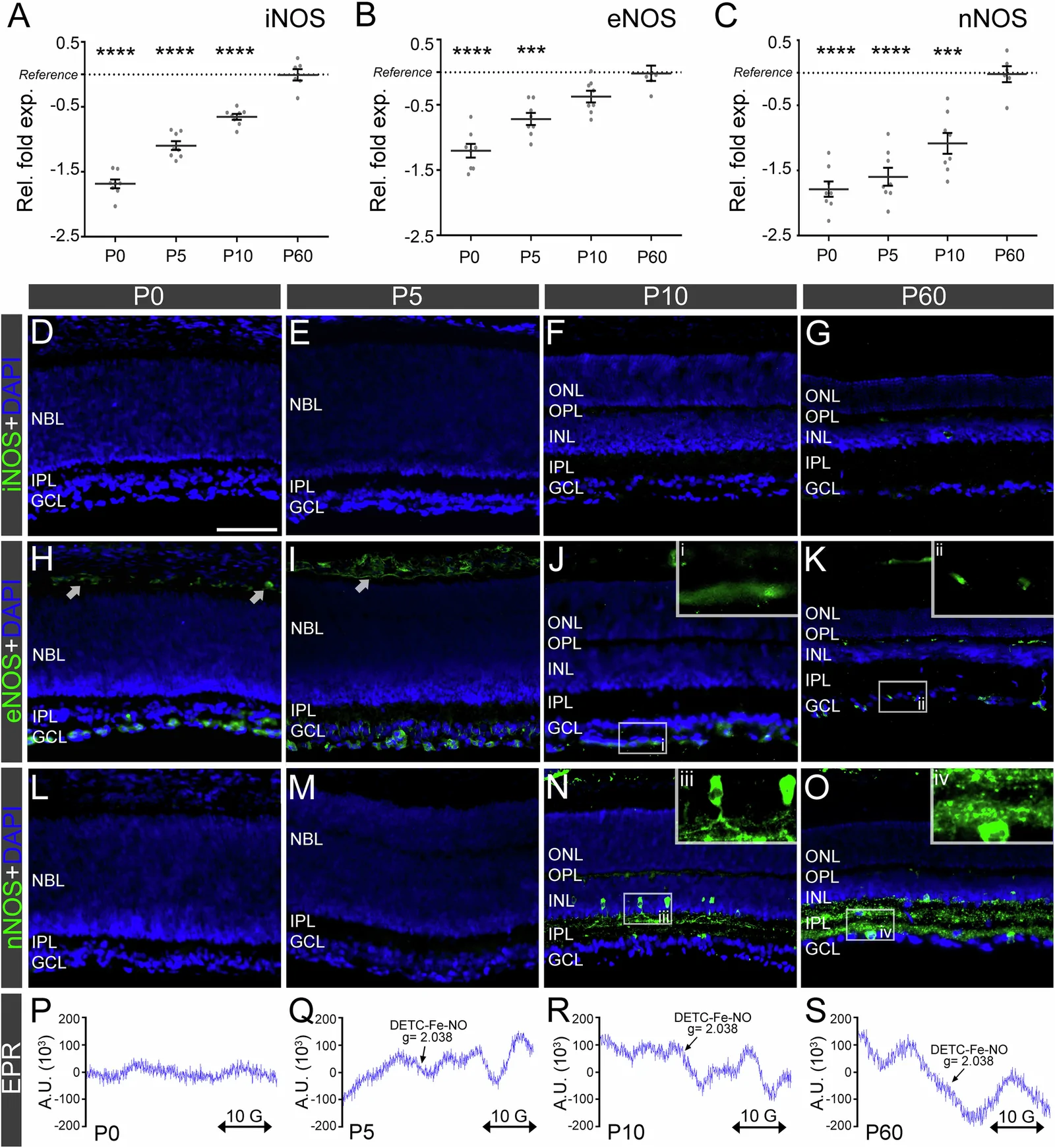
Emergence of nNOS expression and NO production during retinal development. Quantitative real-time PCR analysis of iNOS (**A**), eNOS (**B**), and nNOS (**C**) mRNA expression in retinas from P0, P5, P10, and adult (P60) rats. Expression levels were normalized to P60 values using the ΔΔCt method (2^–ΔΔCt^), followed by log-transformation, and are presented as mean ± SEM (*n* = 8). Individual data points are shown (gray dots). ***p < 0.001, ****p < 0.0001 vs. P60 (one-way ANOVA followed by Tukey’s post hoc test). **D**–**O** Immunofluorescence for iNOS (**D**–**G**), eNOS (**H**–**K**), and nNOS (**L**–**O**) in retinas at the same ages, counterstained with DAPI. Insets highlight eNOS labeling near the ganglion cell layer (GCL) at P10 (**J**, i) and P60 (**K**, ii), and nNOS labeling in the inner nuclear layer (INL) at P10 (**N**, iii) and in the inner plexiform layer (IPL) at P60 (**O**, iv). Arrows indicate eNOS immunoreactivity in the retinal pigment epithelium (RPE). NBL neuroblastic layer, ONL outer nuclear layer, OPL outer plexiform layer. Scale bar: 50 µm. Representative electron paramagnetic resonance (EPR) spectra showing the NO–Fe²⁺-(DETC)₂ signal in P0 (**P**), P5 (**Q**), P10 (**R**), and P60 (**S**) retinas (*n* = 3 per group). Arrows indicate the paramagnetic signal of the NO complex. G Gauss, g microwave frequency.

We initially aimed to understand the role of nitric oxide synthetase (NOS) isoforms during retinal development by analyzing transcript levels and protein distribution. Compared to adult retinas (P60), low levels of all three isoforms were observed. For iNOS, all three ages were lower during development (*F*(3,26) = 106.9, *p* < 0.0001, one-way ANOVA)(fold expression relative to P60: P0: −1.69 ± 0.07, variation of 97.95%, *p* < 0.0001; P5: −1.10 ± 0.07, variation of 91.97%, *p* < 0.0001; P10: −0.65 ± 0.04, variation of 58.68%, *p* < 0.0001, Fig. 1A. eNOS (*F*(3,26) = 24.4, *p* < 0.0001, one-way ANOVA) was lower in P0 and P5 retinas (fold expression relative to P60: P0: −1.21 ± 0.11, variation of 93.50%, *p* < 0.0001; P5: −0.72 ± 0.09, variation of 81.24%, *p* = 0.0004), while in P10 the observed variation was not significant comparing to the adult retina (Fig. 1B). Similar to iNOS, all analyzed ages showed a downregulated nNOS mRNA (*F*(3,26) = 29.27, *p* < 0.0001, one-way ANOVA), which increases during developmental ages (fold expression relative to P60: P0: −1.79 ± 0.12, variation of 98.11%, *p* < 0.0001; P5: −1.60 ± 0.14, variation of 96.74%, *p* < 0.0001; P10: −1.08 ± 0.16, variation of 88.37%, *p* < 0.0001, Fig. 1C).

Regarding protein expression, iNOS was not detected in any evaluated ages (Fig. 1D–G), which could be explained once the physiological environment was maintained. eNOS expression was observed in all analyzed ages, mainly in the retinal pigment epithelium (RPE) and choroid (white arrows), and in the hyaloid plexus, near to GCL (as observed in the digital magnification i and ii, Fig. 1H–K). nNOS labeling was first observed after P10 (Fig. 1L–O), mainly found in the GCL, IPL, inner nuclear layer (INL), and OPL. In the GCL, nNOS-positive cells presented a morphology compatible with ganglion cells. In the inner and outer edges of INL, nNOS morphology suggests that this protein is located in the horizontal and amacrine cells, respectively. Additionally, nNOS-positive amacrine cells present two different morphologies: the first type (type I) shows a greater area and more intense labeling than the second cell type (type II). Moreover, nNOS labeling in IPL had a puncta pattern disposed in horizontal lines. These lines were not restricted to a specific sublamina (ON or OFF), suggesting that nNOS-positive cell dendrites can be involved in both visual processing pathways.

Next, we evaluated NO levels during retinal development, once the presence of nNOS in two amacrine cell subtypes suggests that NO production may arise from distinct retinal circuitries. This is particularly relevant since the presence of NOS isoforms does not guarantee gas production. For this purpose, we employed the spin trap technique to capture the NO free radical generated in the rat retinas. The spin trap Fe(DETC)^2^ forms the stable complex NO–Fe(DETC)^2^, which is identified by electron paramagnetic resonance (EPR) spectra [16–19], and this complex is identified in g-factor equals 2.038 as previously used in our work [20]. The EPR spectra showed no detection of NO production in P0 and P5 retinas (Fig. 1P, Q), but a high level of NO production at P10 and P60 retinas (Fig. 1R, S).

### nNOS-positive amacrine cells show distinct morphology since the initial differentiation

**Fig. 2:**
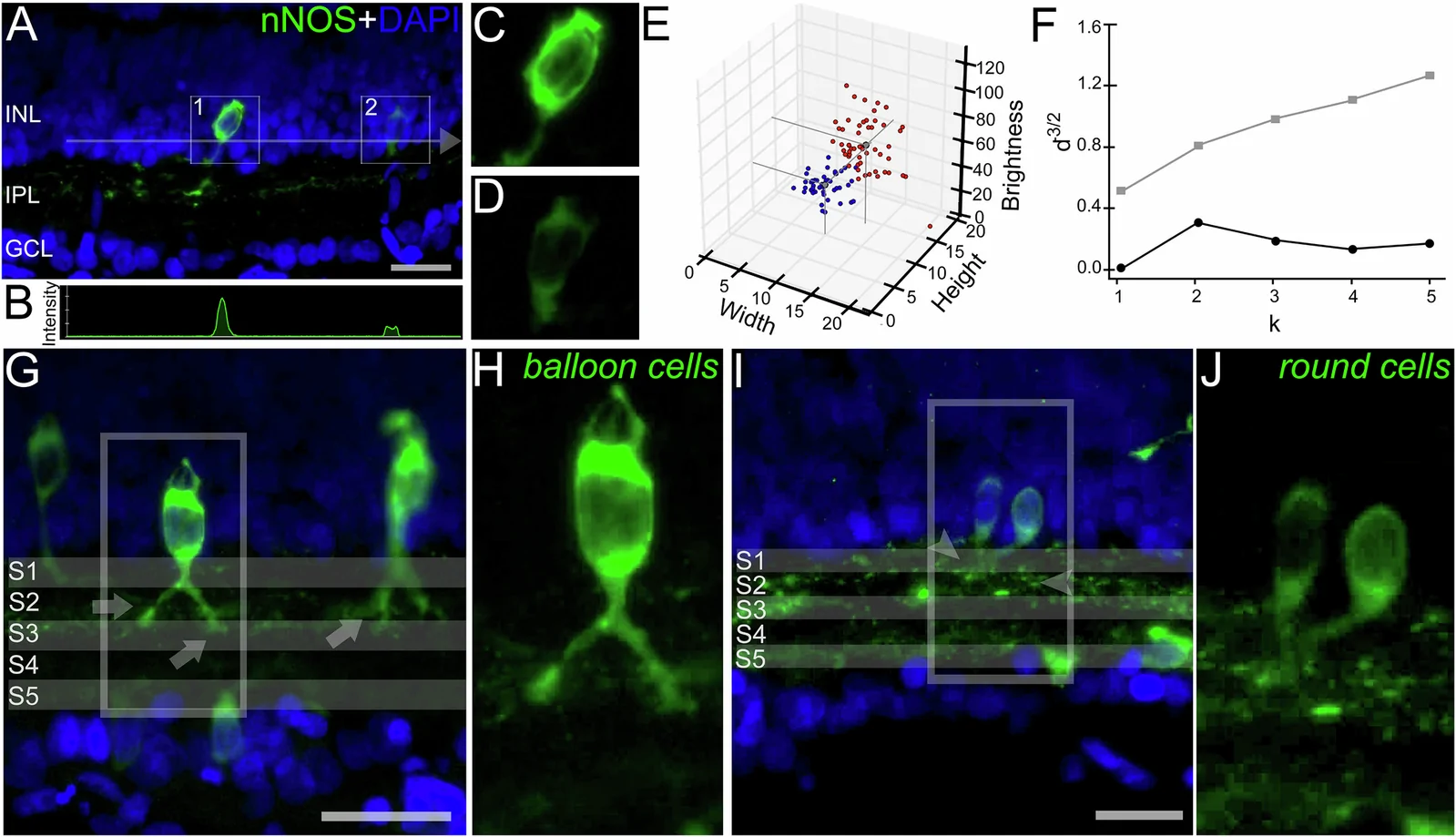
Two morphologically distinct populations of nNOS-expressing amacrine cells in the developing retina. **A** Representative immunofluorescence image of nNOS labeling in a transverse section of P10 rat retina. **B** Pixel intensity profile along the horizontal line shown in (**A**), highlighting areas of high nNOS signal. **C**, **D** High-magnification views of two distinct nNOS+ amacrine cell populations in regions marked in (**A**): round cells (**C**) and balloon-shaped cells (**D**). **E** Three-dimensional scatter plot of individual nNOS+ cells based on morphological features (soma width, brightness, and height relative to the INL–IPL border). Clustering analysis using the k-means algorithm (*k* = 2) revealed two distinct cell populations, with centroids represented by gray dots. **F** Rescaled distortion curve and jump analysis confirming the optimal number of clusters at *k* = 2. **G**–**J** Representative balloon-shaped (**G**, **H**) and round (**I**, **J**) nNOS+ cells, showing distinct stratification patterns within the inner plexiform layer (IPL). Arrows indicate balloon-cell stratification in specific IPL sublaminas; arrowheads indicate stratification of round cells. The IPL was divided into five equal sublayers (S1–S5) for analysis. Nuclei were counterstained with DAPI. INL inner nuclear layer, IPL inner plexiform layer, GCL ganglion cell layer. Scale bar: 25 µm.

Once we identified NO production during retinal development concurrent with nNOS localization into two amacrine cell types, we decided to investigate this isoform in more detail. We previously observed two distinct populations of nNOS-positive amacrine cells in the adult rat retina [20], which were called *round* and *balloon* cells. We hypothesize that P10 retinas also exhibit these differences. To investigate this, we evaluated the parameters of width, brightness, and distance from the inner INL (height) (Fig. 2A–D) in nNOS-positive amacrine cells of the P10 retina (*n* = 6). The values for each parameter for each cell were plotted in a three-dimensional graph (Fig. 2E). Using a clustering method, we obtained a *k* value of 2, where *k* is the number of clusters calculated, confirming the existence of two distinct populations (Fig. 2F). Each cell group (or cluster) is represented in Fig. 2E in blue and red colors.

Due to the morphological features of these two populations, we proposed that the blue-represented cells are the round cells, while the red-represented cells are balloon cells. The average of each parameter was calculated based on clustering data, distinguishing the two populations. At P10, balloon cells had a brightness of 63.02 ± 19.03, a width of 13.29 ± 2.20, and an INL distance of 13.70 ± 2.41, while the round cells showed a brightness of 39.70 ± 1.04, a width of 10.19 ± 0.20, and an INL distance of 9.60 ± 0.31. Furthermore, the IPL stratification appears to differ between the two cell types: balloon cells exhibit ramification in sublaminas 2 and 3 (Fig. 2G, H, indicated by arrows), while round cells mainly stratify in strata 1, 2, and 3 (Fig. 2I, J, indicated by arrowheads).

### Endogenous NO does not modulate retinal waves but affects network connectivity

**Fig. 3:**
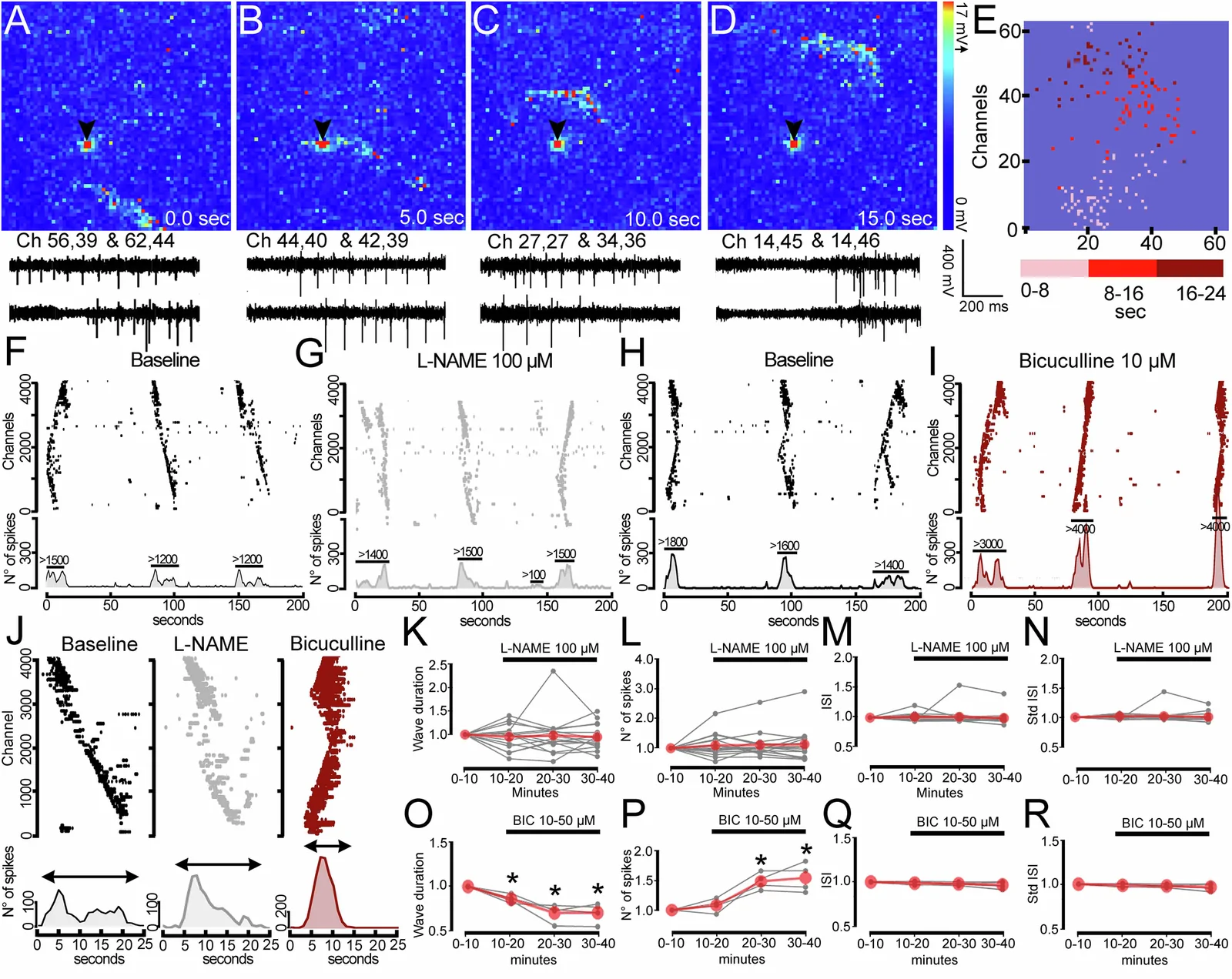
NOS inhibition does not alter Stage III retinal waves recorded by HD-MEA. **A**–**D** Representative color-coded spontaneous activity maps recorded from P11 retinas using a 64 × 64 high-density multielectrode array (HD-MEA). The color scale indicates voltage amplitude (blue = 0 mV; red ≥17 mV) in 500 ms bins. The traces below show simultaneous recordings from two selected channels. Arrowheads indicate representative noise channels. **E** Activated channels during the propagation of a single retinal wave are displayed in three successive 8-s intervals. **F**–**I** Raster plots and quantification of spike counts in control (**F**, **H**), after L-NAME (100 µM; **G**), and bicuculline (10 µM; **I**) application. **J** Raster plots and quantification of spike counts during a single retinal wave in control, L-NAME, and bicuculline conditions. Double-headed arrows indicate wave duration. **K**–**N** Quantitative analysis of Stage III wave parameters under control conditions (0–10 min) and after L-NAME application (10–40 min): **K** wave duration, **L** intra-wave spike number, **M** inter-spike interval (ISI), and **N** ISI standard deviation (Std ISI). **O**–**R** Same analyses as in (**K**–**N**) for recordings performed under bicuculline treatment (10 µM and 50 µM, 10–40 min). Gray traces and inserts show individual normalized data; red traces and inserts represent group means ± SEM. In all plots, *n* = 16 for L-NAME and *n* = 4 for bicuculline-treated retinas. *p < 0.05, one-way ANOVA with multiple comparisons.

**Fig. 4:**
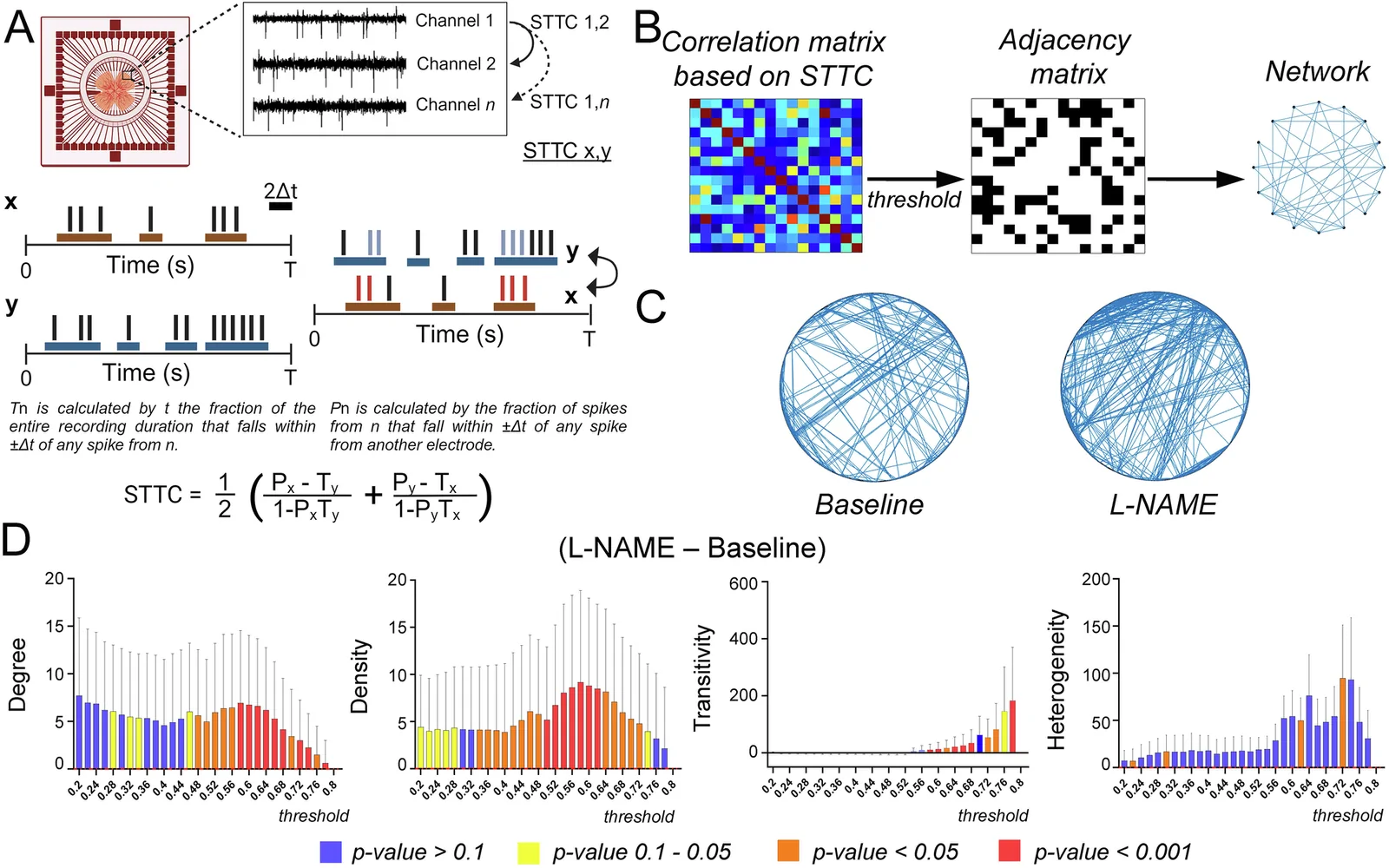
NOS inhibition alters network connectivity in the developing retina. **A** Representative HD-MEA recording of spontaneous activity from a P11 retina. Peak detection was performed to extract binary spike trains from individual electrodes, and the pairwise spike-time tiling coefficient (STTC) was calculated to assess functional connectivity. **B** Correlation matrices were generated from STTC values, and thresholding was applied to obtain adjacency matrices representing network connectivity. **C** Representative graphs illustrating functional networks reconstructed from the same retina before (baseline) and after L-NAME (100 µM) treatment. **D** Quantitative analysis of network graph parameters, including degree, density, transitivity, and heterogeneity, as a function of the applied threshold (*n* = 16). L-NAME treatment significantly increased the degree and density while altering other graph metrics, indicating that NO modulates retinal network topology during development.

Once we observed that nNOS is located in amacrine cells, which are closely related to retinal waves, and considering NOS role during SNC development, as in synaptic pruning and neuronal activity, we decided to investigate whether the NOS pharmacological inhibition interferes with neuronal spike activity. For this purpose, we recorded spontaneous retinal waves, a developmental activity that facilitates neuronal network refinement for retinal receptive fields and visual map formation before the establishment of visual experience [3, 21]. We used an HD-MEA with 4,096 channels (64 ×64, 3Brain, Switzerland). Retinas were recorded under basal conditions and in the presence of NOS inhibition by L-NAME (Fig. 3), which can virtually abolish NO production in a nonspecific manner (Fig. 4A). Our results demonstrate that NOS blockade does not acutely modulate retinal waves or neuronal spike activity (Fig. 3, K-N). To test whether the retinal waves were sensitive to pharmacological intervention, we applied bicuculline to the recording retinas, an antagonist of the GABAA receptor that modulates retinal wave activity [14, 22]. Our results demonstrated that bicuculline reduces wave duration and increases the number of spikes in the waves (Fig. 3, O-P; wave duration: 10–20 min = 0.865 ± 0.016; 20–30 min = 0.688 ± 0.053; 30–40 min = 0.689 ± 0.054; the number of spike intra-wave: 20–30 min = 1.480 ± 0.071; 30–40 min = 1.544 ± 0.120, *P* < 0.05 in one-way ANOVA).

Afterward, the retinal network connectivity was analyzed using spontaneous activity. Binary series were obtained after peak detection and used to determine pairwise correlations with STTC, generating a correlation matrix (Fig. 4A). Next, adjacency matrices and graphs were defined according to different thresholds (Fig. 4B), based on recordings from baseline and L-NAME (100 µM) conditions (Fig. 4C). The network parameters, including mean degree, connection density, transitivity, and heterogeneity, were higher in L-NAME. To quantify the differences, we subtracted the baseline parameter values from those of L-NAME and displayed these differences across various thresholds (Fig. 4D). Compared to the baseline, L-NAME resulted in an increased degree and density, indicating a more connected network. Conversely, L-NAME had only a negligible, non-significant effect on transitivity and heterogeneity (Fig. 4D).

### L-NAME and 7-NI block NO production and regulate the expression of synaptic genes in retinal explants

**Fig. 5:**
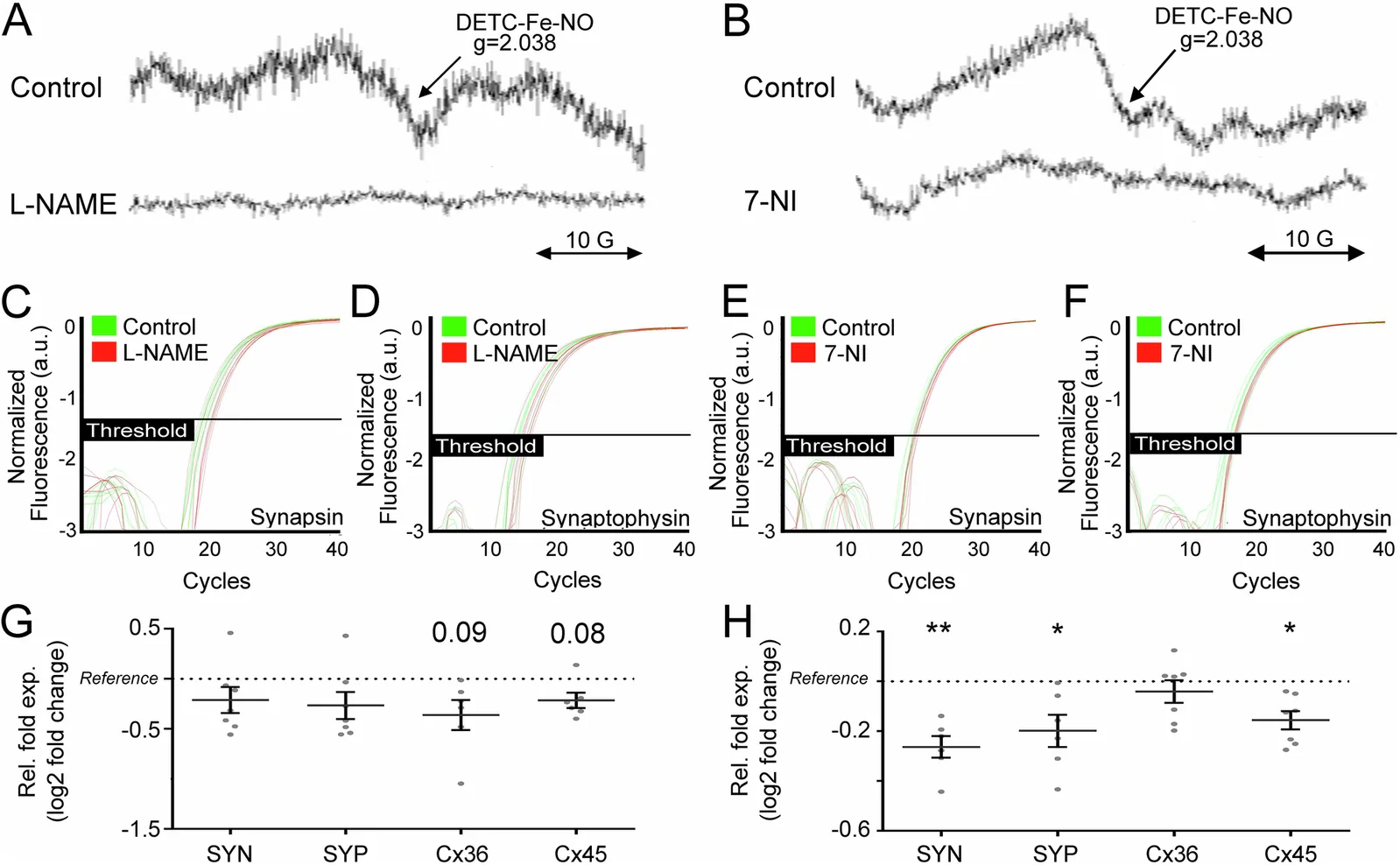
NO production increases during retinal development and regulates synaptic gene expression. **A**, **B** Electron paramagnetic resonance (EPR) spectra showing NO levels in P9–P10 retinal explants. **A** Control explants and explants treated with the non-selective NOS inhibitor L-NAME (100 µM). **B** Control explants and explants treated with the nNOS-selective inhibitor 7-nitroindazole (7-NI, 100 µM). Arrows indicate the paramagnetic signal corresponding to the NO–Fe²⁺-(DETC)₂ complex. **C**–**F** Amplification plots of synapsin (SYN; **C**, **E**) and synaptophysin (SYP; **D**, **F**) transcripts obtained by quantitative PCR from explants treated with L-NAME (**C**, **D**) and 7-NI (**E**, **F**). Quantification of mRNA expression levels of synaptic genes encoding electrical (Cx36, Cx45) and chemical (SYN, SYP) synaptic proteins after treatment with L-NAME (**G**) or 7-NI (**H**). Data were normalized to control values (relative expression set to 20). Values are expressed as mean ± SEM (*n* = 6). *p < 0.05, **p < 0.01 com*p*ared to control (in unpaired t-test). EPR experiments were performed in triplicate (*n* = 3). AU arbitrary units, G Gauss, g microwave frequency; Cx connexin.

Once we observed that L-NAME did not alter the electrophysiological signature but did affect the retinal connectivity pattern, we aimed to investigate whether NO blockade could impact retinal development and synapse formation at the molecular level. To this end, we used two NO pharmacological blockers: L-NAME (as shown above) and 7-NI, a specific nNOS inhibitor. Retinal explants from P9 mice were treated, and NO production was assessed using EPR spectroscopy. EPR confirmed that broad-spectrum L-NAME and nNOS-specific 7-NI inhibitors reduced NO production in developing retinal explants (Fig. 5A, B).

After validating both inhibitors by EPR, we assessed the expression of genes involved in chemical and electrical synapses in P9-10 rat retinal explants following NOS blockade (Fig. 5G–L). We observed that L-NAME treatment does not affect the chemical synapses (*p* > 0.05 for both SYN and SYP, paired t-test), as well as the connexins; although the levels of connexins showed a tendency to decrease comparing with the control (Cx36: relative fold expression −0.36 ± 0.15, variation of 48.17%, *t*(10) = 1.831, *p* = 0.0970, unpaired t-test; Cx45: relative fold expression −0.22 ± 0.08, variation of 37.14%, *t*(10) = 1.934, *p* = 0.0819 in unpaired t-test, Fig. 4I). On the other hand, specific nNOS inhibition using 7-NI seems have a more impact on synaptic genes, downregulating SYN (relative fold expression −0.26 ± 0.04, variation of 46.39%, *t*(9) = 2.753, *p* = 0.0058 in unpaired t-test), SYP (relative fold expression −0.19 ± 0.06, variation of 38.15%, *t*(9) = 2.753, *p* = 0.015 in unpaired t-test) and Cx45 (relative fold expression −0.13 ± 0.04, variation of 26.10%, *t*(12) = 2.504, *p* = 0.0277 in unpaired t-test). Cx36 did not show differences compared to the control (*p* > 0.05, Fig. 5L).

### Neuronal branching is changed by nNOS inhibitor

**Fig. 6:**
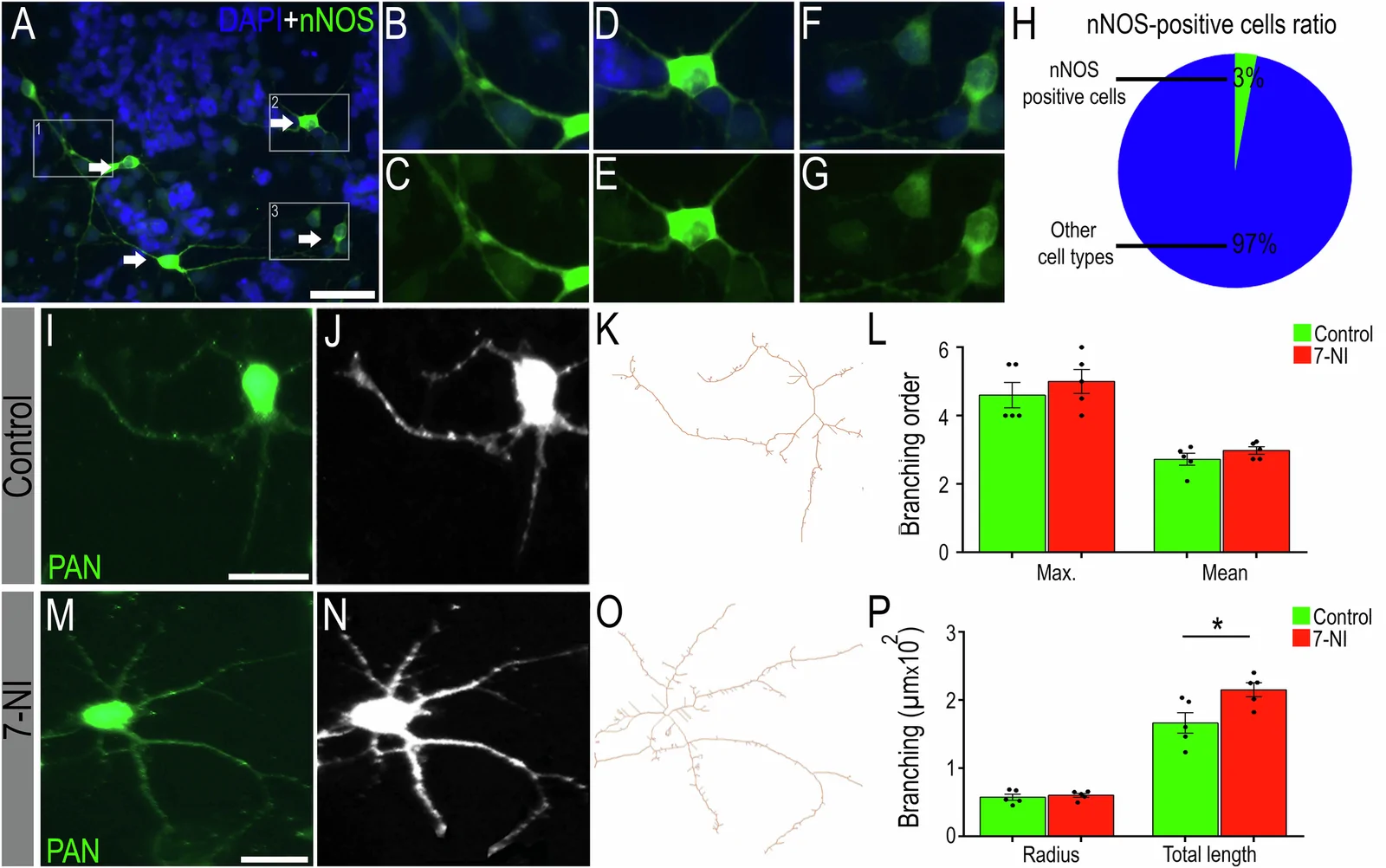
nNOS-positive cells in retinal primary cultures and the effects of nNOS inhibition on neurite growth. **A** Immunofluorescence of retinal primary cultures showing nNOS-positive cells (arrows) counterstained with DAPI. Scale bar: 50 µm. **B**, **C** Representative images showing the connectivity of nNOS-positive cells in vitro. **D**–**G** Identification of two distinct populations of nNOS-positive cells differing in soma size and fluorescence intensity. **H** Quantification of the ratio between nNOS-positive and nNOS-negative cells (*n* = 4 independent cultures). **I**–**P** Effect of nNOS inhibition on neurite growth assessed by treating cultures with 7-nitroindazole (7-NI, 100 µM). Control cultures (vehicle-treated): neurons labeled with PAN antibody (green; **I**), corresponding black-white mask for neurite visualization (**J**), and skeletonized image used for morphological analysis (**K**). Cultures treated with 7-NI showing neurons labeled with PAN (**M**), masked (**N**), and skeletonized images (**O**) as above. Scale bar: 20 µm. **L** Quantification of branching order (maximum and mean values). **P** Quantification of branching radius and total neurite length (expressed as µm × 10³). Data are presented as mean ± SEM. *p < 0.05 (unpaired t-test; *n* = 5).

Since L-NAME did not acutely modulate retinal waves during development, and induced only minor changes in gene expression, while the nNOS-specific inhibitor 7-NI significantly altered synaptic gene expression, we proceeded to investigate the impact of 7-NI on neurogenesis, both in vitro and in vivo. First, we intended to observe nNOS labeling in retinal primary cell culture (Fig. 6A–H) and measure neuronal branching (Fig. 6I–P). We first observed that in vitro nNOS presents two distinct cell populations, both with a predominantly cytoplasmic localization (Fig.6D–G), similar to *round* and *balloon* cells observed in the retinal tissue. We observed that nNOS-positive cells represent approximately 3.60 ± 0.03% of total seeded cells (Fig. 6H), and those cells seem to establish synaptic connections among each other (Fig. 6B, C). Once we confirmed the presence of nNOS-positive cells in vitro, we sought to determine whether NO produced by nNOS could influence neuronal branching. With this proposal, we treated the cells with 7-NI (10 µM; *n* = 5) and observed the neuritogenesis (Fig. 6I–P). Using the Trees Toolbox and MATLAB, we demonstrated no differences in maximum branching order and mean branching order (*p* > 0.05) (Fig. 6L). Similarly, no change was noticed in the neurite radius (*p* > 0.05) (Fig. 6P). On the other hand, the neurites’ total length was increased after 4 days of treatment (166.50 mm ± 15.22 mm vs. 215.10 mm ± 10.28 mm, Ctl vs. 7-NI, *t*(8) = 2.65, *p* = 0.0292 in unpaired t-test) (Fig. 6P). In summary, our results suggest that inhibiting NO production alters the number of diverse neurofilament levels, without affecting neurite developmental levels or neurite radius, but alters the total neurite length.

### Inhibition of nNOS activity regulates the accumulation of synaptic proteins in vivo

**Fig. 7:**
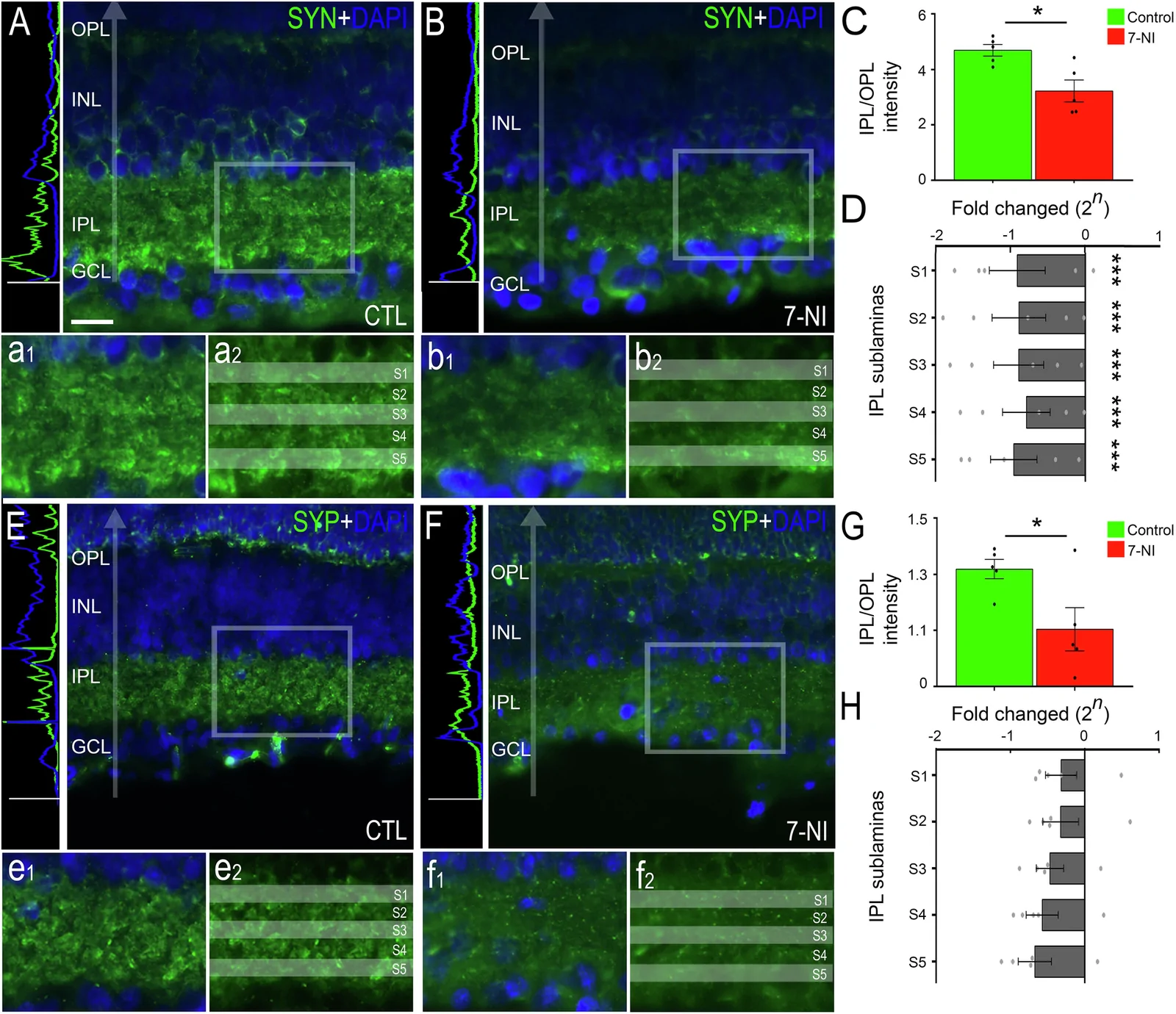
nNOS inhibition disrupts the laminar distribution of chemical synaptic markers in vivo. Immunofluorescence images and corresponding pixel intensity profiles of Synapsin (SYN) labeling in retinal sections 24 h after subretinal injection of vehicle (**A**) or 7-nitroindazole (7-NI; 1 mM, **B**). Insets show higher magnification of selected regions. **C** IPL/OPL fluorescence intensity ratio of SYN labeling. **D** Normalized intensity profile of SYN across IPL sublayers (S1–S5) in treated retinas relative to control. Immunofluorescence images and corresponding pixel intensity profiles of Synaptophysin (SYP) labeling after subretinal injection of vehicle (**E**) or 7-NI (**F**), with higher magnification of selected areas. **G** IPL/OPL fluorescence intensity ratio of SYP labeling. **H** Normalized intensity profile of SYP across IPL sublayers (S1–S5) in treated retinas relative to control. For all panels, the IPL was subdivided into five equal sublayers (S1–S5). Nuclei were counterstained with DAPI. OPL outer plexiform layer, INL inner nuclear layer, IPL inner plexiform layer, GCL ganglion cell layer. Scale bar: 50 µm. Data are presented as mean ± SEM (*n* = 5). *p < 0.05 (paired t-test for **C**, **G**); ***p < 0.001 (two-way ANOVA followed by Sidak’s post hoc test for **D**, **H**).

**Fig. 8:**
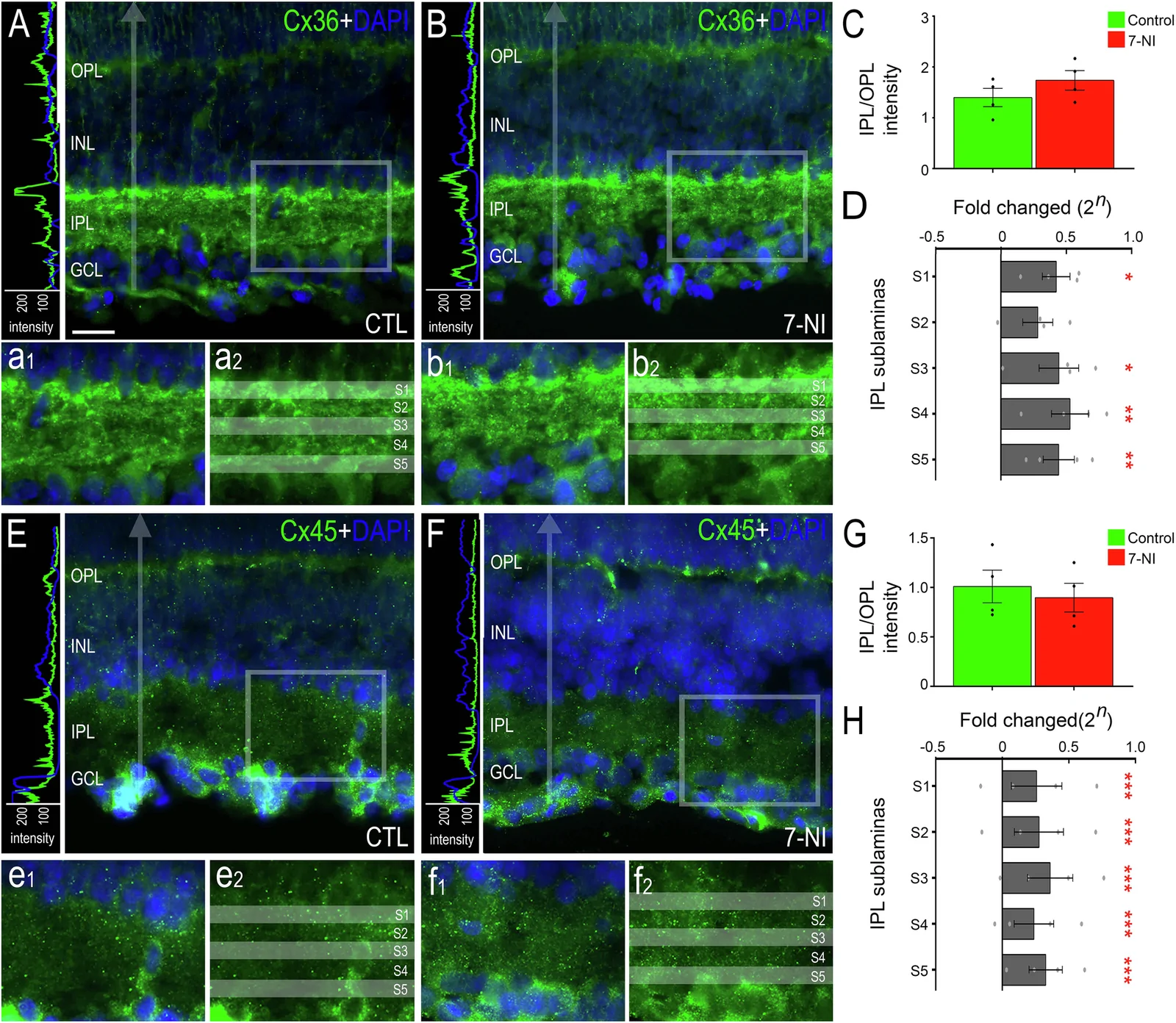
nNOS inhibition disrupts the laminar distribution of electrical synaptic markers in vivo. Immunofluorescence images and corresponding pixel intensity profiles of connexin-36 (Cx36) labeling in P11 retinal sections, 24 h after subretinal injection of vehicle (**A**) or 7-nitroindazole (7-NI, 1 mM; **B**). Insets show high-magnification views of selected IPL regions. **C** IPL/OPL fluorescence intensity ratio of Cx36 labeling. **D** Normalized intensity profile of Cx36 signal across the IPL sublayers (S1–S5) in treated retinas relative to control. Immunofluorescence images and pixel intensity profiles of connexin-45 (Cx45) in control (**E**) and 7-NI-treated (**F**) retinas. **G** Normalized intensity profile of Cx45 across IPL sublayers (S1–S5) after treatment. In all images, the IPL was subdivided into five equal sublayers (S1–S5) for analysis. Nuclei were counterstained with DAPI. OPL outer plexiform layer, INL inner nuclear layer, IPL inner plexiform layer, GCL ganglion cell layer. Scale bar: 50 µm. Data are expressed as mean ± SEM (*n* = 5). *p < 0.05, **p < 0.001, ***p < 0.001 (two-way ANOVA followed by Sidak’s post hoc test in **D**, **G**).

Since 7-NI was not toxic to the retina in vivo, as shown by the TUNEL assay (data not shown), we performed a subretinal injection of 7-NI (1 mM). We evaluated the synaptic markers through the pixel profile pattern, IPL/OPL ratio intensity, and the intensity in each IPL sublayer (S1–S5, Figs. 7 and 8). All analyses were based on the mean intensity. Our results indicate that levels of chemical synaptic proteins were generally reduced following 7-NI treatment. SYN (*n* = 5) showed a general decrease in the intensity IPL/OPL ratio (approximately 32.08%, representing 3.52 ± 0.16 vs. 2.42 ± 0.30, vehicle vs. 7-NI, *t*(4) = 2.936, *p* = 0.0425 in paired t-test, Fig. 7A–C). SYN intensity in all IPL sublaminas was also reduced after NO inhibition (S1: 2^−0.91^, S2: 2^−0.89^, S3: 2^−0.89^, S4: 2^−0.79^, S5: 2^−0.95^, fold-expression 2^*n*^, (*F*(4, 16) = 24.87, *p* < 0.0001, two-way ANOVA), Fig. 7D). SYP also showed an IPL/OPL reduction (1.32 ± 0.03 vs. 1.10 ± 0.08, vehicle vs. 7-NI, *t*(4) = 3.054, *p* = 0.0379 in paired t-test, Fig. 7G). Still, the sublaminas did not differ from the control group (*p* > 0.05), possibly due an outlier between the samples (Fig. 7H).

Electrical synaptic proteins, on the other hand, seem to behave differently. Both Cx36 and Cx45 proteins did not exhibit an altered IPL/OPL intensity ratio after 7-NI treatment (*p* > 0.05) (Fig. 7C, G). However, when the IPL sublaminas were individually compared, most of them show a significant overexpression when compared to control retinas: Cx36 shows an increase in S1, S3, S4, and S5 sublayers (S1: 2^0.42^, S3: 2^0.44^, S4: 2^0.53^ and S5: 2^0.44^, fold-expression 2^*n*^, *p* = 0.0243, *p* = 0.0104, *p* = 0.0013 and *p* = 0.0034, respectively, Sublamina factor = *F* (4, 12) = 6.403, *p* = 0.0054, Fig. 6D). At the same time, Cx45 was generally upregulated after treatment (S1: 2^0.26^, S2: 2^0.28^, S3: 2^0^^.36^, S4: 2^0.24^, S5: 2^0.33^, fold-expression 2^*n*^, *p* < 0.001, Fig. 6H).

## Discussion

The assembly of functional neural circuits depends on the coordinated action of genetic programs, spontaneous neuronal activity, and molecular signals [2, 23]. By integrating EPR spectroscopy, HD-MEA recordings with 4096 channels, and graph-theoretical analysis, we reveal a previously unrecognized role of NO in modulating retinal circuit formation. Our findings demonstrate that NO, produced by nNOS-expressing amacrine cells, does not significantly influence the spatiotemporal dynamics of Stage III retinal waves; however, it critically regulates synaptic gene expression, network connectivity, and the laminar distribution of synaptic proteins. These results position NO as a key molecular signal that refines retinal circuits through mechanisms that are largely independent of the spatiotemporal properties of retinal waves, a concept that has been less frequently demonstrated in developing sensory networks.

The emergence of NO during the period of intense synaptogenesis in the developing retina is temporally aligned with the maturation of Stage III retinal waves [3, 14, 24]. NO has been implicated in regulating topographic map refinement in the retinocollicular and retinogeniculate pathways [15] and in synaptogenesis in other regions, such as the hippocampus [25] and cerebellum [6]. In the retina, previous studies have shown that ambient conditions and activity can regulate connexin and synaptic protein expression [26, 27]. Yet whether NO modulates these processes in the retina and whether such modulation requires spontaneous activity remained unknown. By combining EPR spectroscopy, HD-MEA recordings, and graph-theoretical analysis, we demonstrate that NO is dispensable for spontaneous activity patterns but is essential for regulating network structure and synaptic composition.

Interestingly, NOS inhibition increased network degree and density, indicating a shift toward more densely connected but potentially less selective circuits. This pattern may reflect reduced synaptic specificity during circuit refinement, leading to increased redundancy and decreased precision of connectivity. Similar observations have been reported in developmental models in which inhibitory constraints are reduced, resulting in hyperconnected yet functionally immature networks [12]. In this context, NO may act as a modulatory signal that constrains excessive synaptic connectivity, thereby fine-tuning the emerging network architecture. Importantly, these topological changes are not merely structural: computational modeling indicates that specific patterns of degree correlations can influence network sensitivity to subthreshold inputs [28], suggesting that the altered graph structure observed here may have direct implications for information processing in the developing retina.

The identification of two morphologically distinct nNOS-positive amacrine cell populations is noteworthy. These populations exhibit specific laminar patterns within the IPL, suggesting that they may differentially modulate distinct retinal microcircuits [29, 30]. Given that the laminar-specific distribution of synapses is essential for the emergence of functionally specialized visual pathways [31], our findings implicate NO in this stratification process.

At the molecular level, the downregulation of SYN, SYP, Cx36, and Cx45 following nNOS inhibition reveals a broad impact of NO on both chemical and electrical synaptogenesis. This finding is consistent with evidence from other systems, which shows that NO regulates the expression of synaptic proteins and gap junction components [32, 33]. Moreover, our data indicate that NO constrains neurite outgrowth, as nNOS inhibition increased neurite length without affecting branching complexity, in line with studies in hippocampal and cortical neurons [25]. It is important to note that our conclusions are based on pharmacological inhibition, which inherently carries limitations, including potential off-target effects and limited cell-type specificity. Future studies using genetic approaches will be necessary to further dissect cell-type-specific contributions and validate the mechanisms described here.

Importantly, our use of graph-theoretical approaches allowed us to detect subtle alterations in network topology that could not be captured by traditional activity analysis alone. Recent studies have highlighted the importance of graph analysis in describing functional and structural changes in developing neural circuits [34, 35], reinforcing the value of this methodology in detecting early circuit remodeling.

Understanding how NO shapes neural circuits may offer new insights into the pathophysiology of neurodevelopmental disorders associated with impaired synaptic organization, including autism spectrum disorders and certain developmental epileptic encephalopathies [36, 37]. Our results suggest a potential mechanistic link between altered NO signaling and the improper assembly of retinal synaptic networks, raising the possibility that similar pathways, at least partially dissociable from patterned spontaneous activity, may operate in other brain regions during development.

In summary, we demonstrate that NO, released by nNOS-expressing amacrine cells, plays an essential role in shaping retinal synaptic networks during development by modulating synaptic gene expression, neurite growth, and network topology, independently of spontaneous activity patterns. This work reveals an activity-independent molecular mechanism for circuit refinement, expands the functional repertoire of NO in neural development, and offers new conceptual and translational perspectives for neurodevelopmental disorders characterized by impaired synaptic organization.

## Materials and methods

### Animal care and experimental model

All experiments were conducted following the guidelines of the National Institutes of Health and the Brazilian Society for Neuroscience and Behavior. The experimental protocols were approved by the Animal Research Ethics Committee (CEUA) of UFABC (protocol #16/2014). Both male and female Wistar rats were utilized and housed under controlled conditions, including a 12-h light/dark cycle, 22 °C, and ad libitum access to food and water.

### Animal procedures

Experiments were conducted using Long-Evans rats (*Rattus norvegicus*) aged 0–60 postnatal days (P0-P60) of both sexes. The animals were maintained on a 12-h light/dark cycle (light phase: 80–100 lx), with the lights on at 7:00 a.m. The animals were euthanized with a lethal dose of Urethane (1 g/kg, #101416692, Sigma, St. Louis, MO, USA), and the retinas were carefully dissected to preserve their integrity.

For in vivo nNOS blockade, P9 animals were anesthetized with isoflurane, and 4 µL of 7-nitroindazole 1 mM (7-NI; #N7778, Sigma, St. Louis, MO, USA) was injected into the subretinal space using a Hamilton syringe. The vehicle (5% DMSO in PBS 0.9%) was injected into the contralateral eye. After the injections, the animals returned to a 12-h light/dark cycle. The following day, P10 rats were euthanized, and the retinas were dissected for various analyses.

### Retinal explant preparation and treatment

Retinal explants were obtained from P0, P5, P10, P11, and P60 rats. After decapitation, the eyes were dissected in ice-cold artificial cerebrospinal fluid (ACSF) containing (in mM): 124 NaCl, 3 KCl, 1.25 KH_2_PO_4_, 2 MgSO_4_, 2 CaCl_2_, 26 NaHCO_3_, and 10 glucose, pH 7.4, oxygenated with 95% O_2_/5% CO_2_. Retinas were maintained in a net submerged in oxygenated BME buffer (Neurobasal-A supplemented with 5% FBS, 1% L-glutamine) in vitro for 24–48 h in culture medium (Neurobasal-A with B27 supplement, 0.5 mM GlutaMAX, and antibiotics). For pharmacological treatments, explants were exposed to either vehicle (control) or to the NOS inhibitors L-NAME (100 µM) or 7-Nitroindazole (7-NI, 100 µM), which were added to the culture medium.

### Quantitative PCR (qPCR)

Total RNA was extracted from retinal explants with TRIzol reagent (Invitrogen) and reverse-transcribed into cDNA. qPCR was conducted using SYBR Green Master Mix (Applied Biosystems) and gene-specific primers for nNOS, eNOS, iNOS, Cx36, Cx45, SYN, and SYP (Table 1). Gene expression was normalized to GAPDH quantified using the ΔΔCt method (2^–ΔΔCt^), followed by log-transformation.

**Table 1:** Primers designed for this study.

Gene	Forward primer	Reverse primer
iNOS	CCGCCAAGCTGAACTTGAGT	CATGATAACGTTTCTGGCTCTTGA
nNOS	CCAATGTTCACAAAAAACGAGTCT	TCGGCTGGACTTAGGGCTTT
eNOS	CCAATGTTCACAAAAAACGAGTCT	TCGGCTGGACTTAGGGCTTT
SYN	GCCACCACCCATCATTGCTGC	GGGCTGGCTCTGGAAGGTTGAA
SYP	CGGGCCTCAGGGCGACTATG	GCCCACCCGTGGGATCTTCA
Cx36	GGGGAATGGACCATCTTGGA	TCCCCTACAATGGCCACAAT
Cx45	GGGTAACGGAGGTTCTGGTT	AAGCTCCAACTCATGGTGGT
Cyclophilin	GCGTTTTGGGTCCAGGAATGGC	TTGCGAGCAGATGGGGTGGG

### EPR spectroscopy

NO production was quantified using EPR with Fe^2+^-(DETC)_2_ spin trapping as previously described [20]. Retinal explants were incubated into freshly prepared 1 mL solution of 25 mM HEPES buffer pH 7.4 containing 500 mM Fe(SO4)2 and 500 mM of N,N-diethylditylcarbamate (DETC) for 30 min at 37 °C, frozen in liquid nitrogen, and analyzed using a Bruker ESP300 spectrometer (Bruker, Germany).

### Primary retinal cell culture

Retinal cells from P0 rats were enzymatically dissociated using papain (15 U/mL; Sigma-Aldrich) and subsequently mechanically triturated. Cells were plated at a density of 1 × 10^5^ cells/cm² onto poly-D-lysine-coated coverslips and maintained in Neurobasal-A medium supplemented with B27, FBS, GlutaMAX, and antibiotics. Cultures were kept for 7–10 days in vitro (DIV) and treated with 7-NI (100 µM) or vehicle at DIV 6 for 24 h before analysis.

### Immunofluorescence

Immunolabeling was conducted on fixed retinal sections and cell cultures using primary antibodies against nNOS, SYN, SYP, Cx36, Cx45, and corresponding fluorescent secondary antibodies (Table 2). Images were acquired using a fluorescent Nikon TS100F inverted microscope (Nikon Instruments Inc., Melville, NY, USA). For the retinal sections, the IPL was divided into five equal strata (S1–S5) for laminar analysis.

**Table 2:** Antibodies used in this study.

Antibody	Supplier	Cat #	IF in vivo	IF in vitro
nNOS	Sigma	N7280	1:800	1:5000
eNOS	BD Transduction	610297	1:200	-
iNOS	Millipore	06-573	1:200	-
SUN	Millipore	AB1543	1:300	-
SYP	Millipore	MAB329	1:300	-
Cx36	Invitrogen	36-4600/ 37-4600	1:100	-
Cx45	Millipore	AB1745	1:100	-
Cx43	Invitrogen	71-0700	1:50	-
NeuroChroma-PAN	Millipore	MAB2300	-	1:1000
Secondary antibodies conjugated with Alexa488	Invitrogen	-	1:500	1:3000

### Cluster analysis

From P10 retinal images, we measured three distinct parameters of nNOS-positive cells: brightness, width, and distance to the inner border of the inner nuclear layer as used in previous work [20]. In summary, these attributes were plotted in a three-dimensional graph, where each cell corresponds to a data point. The data were clustered around two distinct centers, which could be identified using *k*-means clustering analysis. The number of clusters, *k*, was calculated by applying the rate-distortion theory proposed by Sugar and James (2003) [38]. In this method, the algorithm finds the clusters and their corresponding centers by minimizing the *d* distortion (*d*) is the sum of squared distances between the cluster centers). The result is observed as a distortion curve *d*(*k*) for different values of *k* generated by the k-means algorithm. The distortion curve is rescaled by the negative power –p/2, based on the dimensionality *p* of the dataset. In our three-dimensional dataset, *d*(*k*) → *d*^−3/2^(*k*).

### Branching analysis

For branching analysis, the NeuroChroma Pan marker (Table 2) was used to examine the potential effects of the nNOS blocker on neuronal arborization. First, cell culture images were pre-processed using the “SynD” toolbox (http://www.johanneshjorth.se/SynD/SynD.html). This toolbox identifies the soma and neurites, creating a clean binary image of the neurons. From this software, the neurite radius and total length of treated retinal neurons were evaluated. Secondly, computational branching analysis was performed by using “Trees Toolbox” (www.treestoolbox.org), an open-source software package written in Matlab (Mathworks, Natick, MA, USA), which provides tools for analyzing, manipulating, and generating the dendritic structure from fluorescent image stacks [39]. Using these computational tools, we were able to investigate the effects of the nNOS blocker on parameters such as maximum and median branching order, which were measured with a default length of 10 µm supplied by the software. Color-coded pictures were generated using the Skeleton tool in ImageJ software to better visualize the branching order. Neurite radius, total length maximum, and median branching order of Ctl vs 7-NI treated cultures were analyzed using Student’s T-Test with a significance level set at 5%.

### Retinal waves recording

P9-11 Long-Evans rats were submitted to rapid eye enucleation after decapitation. Retinas were isolated in aCSF solution containing (in mM) 126 NaCl, 26 NaHCO_3_, 10 D-glucose, 1.45 NaH_2_PO_4_, 2.5 KCl, 1 MgCl_2_, 2 CaCl_2,_ continually oxygenated (5% CO_2_–95% O_2_), and placed on a nitrocellulose membrane (#AABP04700, Millipore, USA) with the ONL facing the membrane. Retinas were kept oxygenated at room temperature (~25 °C) for at least one hour before electrophysiological recordings. For MEA recordings, retinas were placed over the MEA with the RGC layer facing the electrodes and maintained in position by a homemade harp. Retinas were maintained for ~1 h with continuous perfusion of oxygenated aCSF (~2 mL/min at 33 °C) until the spontaneous activity reached stability. Using a high-resolution MEA system consisting of 4096 active electrodes in a 5.12 × 5.12 mm area with an inter-electrode space of 81 µm (BioChip 4096 3Brain GmbH, Switzerland), 10 min recordings were obtained of stable retinal activity (baseline) followed by 30 min recordings with an L-NAME or bicuculline diluted in the aCSF (100 µM or 10–50 µM, respectively). All records were acquired at 7062.1 Hz and saved on hard drives for further analysis.

### Retinal waves analysis

All recordings were submitted for offline analysis using Herding Spikes 2, a highly efficient spike detection method that uses C++ and Python routines [40]. Only channels with more than 5 and fewer than 300 spikes were considered to characterize retinal waves. For each channel, only spikes in a burst were used, which are characterized by sequences of at least 4 events with inter-spike intervals (ISI) less than 1.0 s, whereas an ISI of >1 s denoted the end of the burst. After that, for each 1-s window, the number of spikes in all channels was counted. The retinal wave starts when at least 4 spikes are counted in a row for 5 s and ends when fewer than 4 spikes are counted in a one-second window. Waves with fewer than 50 spikes were discarded. The burst analysis algorithm provided the wave duration, the number of spikes per second, the mean interspike interval (ISI), and the standard deviation of ISI within each wave.

### Graph analysis of retinal networks

Spike detection from MEA recordings generated binary series for each electrode, which were used to determine pairwise correlations using spike time tiling coefficient (STTC), as previously described [41]. The time window used for all data was Δ[1] *t* = 10 ms. Next, a correlation matrix was calculated, from which different adjacency matrices can be generated depending on the correlation threshold. Adjacency matrices and graphs were generated by varying the threshold from 0.2 to 0.8, allowing the calculation of mathematical graph parameters, such as degree, density, transitivity, and heterogeneity. To compare between experimental groups, we evaluated the relative difference of all parameters [42].

For the parameters evaluation, we used “The Brain Connectivity Toolbox” (brain-connectivity-toolbox.net). It is based on MATLAB, and more details can be found in Rubinov et al. [43]. Degree: mean degree per network. Density: number of present connections per possible connections per network. Transitivity: It is the ratio of ‘triangles to triplets’ in the network. Heterogeneity index: a network measure ranging from 0 to 1. If the index is 0, the network is homogeneous (i.e., all nodes are connected to all others); if the index is 1, the network is heterogeneous, resembling a star network [44].

### Statistical analysis

Data are expressed as mean ± SEM. Normality was assessed using the Shapiro-Wilk test. Parametric data were analyzed using Student’s t-test or ANOVA, followed by Tukey’s post hoc test, as appropriate. Graphs were generated using GraphPad Prism 9. A significance level of *p* < 0.05 was adopted.

## Data Availability

MEA recordings are available on https://zenodo.org/records/20148989. Other datasets generated during and/or analyzed during the current study are available from the corresponding author on reasonable request.
